# Metabolic Adaptations May Counteract Ventilatory Adaptations of Intermittent Hypoxic Exposure during Submaximal Exercise at Altitudes up to 4000 m

**DOI:** 10.1371/journal.pone.0049953

**Published:** 2012-11-14

**Authors:** Martin Faulhaber, Tobias Dünnwald, Hannes Gatterer, Luciano Bernardi, Martin Burtscher

**Affiliations:** 1 Department of Sport Science, University Innsbruck, Innsbruck, Austria; 2 Department of Internal Medicine, University of Pavia and IRCCS San Matteo, Pavia, Italy; 3 Folkhälsan Institute of Genetics, Folkhälsan Research Center, University of Helsinki, Helsinki, Finland; University of Las Palmas de Gran Canaria, Spain

## Abstract

Intermittent hypoxic exposure (IHE) has been shown to induce aspects of altitude acclimatization which affect ventilatory, cardiovascular and metabolic responses during exercise in normoxia and hypoxia. However, knowledge on altitude-dependent effects and possible interactions remains scarce. Therefore, we determined the effects of IHE on cardiorespiratory and metabolic responses at different simulated altitudes in the same healthy subjects. Eight healthy male volunteers participated in the study and were tested before and 1 to 2 days after IHE (7×1 hour at 4500 m). The participants cycled at 2 submaximal workloads (corresponding to 40% and 60% of peak oxygen uptake at low altitude) at simulated altitudes of 2000 m, 3000 m, and 4000 m in a randomized order. Gas analysis was performed and arterial oxygen saturation, blood lactate concentrations, and blood gases were determined during exercise. Additionally baroreflex sensitivity, hypoxic and hypercapnic ventilatory response were determined before and after IHE. Hypoxic ventilatory response was increased after IHE (p<0.05). There were no altitude-dependent changes by IHE in any of the determined parameters. However, blood lactate concentrations and carbon dioxide output were reduced; minute ventilation and arterial oxygen saturation were unchanged, and ventilatory equivalent for carbon dioxide was increased after IHE irrespective of altitude. Changes in hypoxic ventilatory response were associated with changes in blood lactate (r = −0.72, p<0.05). Changes in blood lactate correlated with changes in carbon dioxide output (r = 0.61, p<0.01) and minute ventilation (r = 0.54, p<0.01). Based on the present results it seems that the reductions in blood lactate and carbon dioxide output have counteracted the increased hypoxic ventilatory response. As a result minute ventilation and arterial oxygen saturation did not increase during submaximal exercise at simulated altitudes between 2000 m and 4000 m.

## Introduction

Acute high-altitude exposure reduces maximal oxygen uptake and submaximal aerobic endurance performance [1]–[3]. However, chronic high-altitude exposure for days or weeks enhances endurance performance at high altitude partly by ventilatory acclimatization [3]–[5] but also metabolic adaptations seem to be involved [6]. Ventilatory acclimatization results in an increase in minute ventilation and an accompanied improvement in arterial oxygen saturation (SaO_2_) during exercise [3]. The increase in hypoxic ventilatory response (HVR) is an independent and essential mechanism of this process [7] and even short-term exposures to hypoxia augment HVR [8]. Metabolic adaptations during a longer lasting high-altitude exposure lead to reduced blood lactate concentrations (LA) during submaximal exercise and seem not to be caused by an improved oxygen delivery or oxygen utilisation [6].

Intermittent hypoxia exposure (IHE) is defined as repeated passive exposures to hypoxia interspersed by normoxic periods [9]. IHE has been shown to induce altitude acclimatization and therefore might be an effective pre-acclimatization strategy to improve endurance performance during an acute high-altitude exposure [10]–[12].

IHE, applying hypoxic short-term (≤1 hour) exposures, has been repeatedly shown to increase HVR [13]–[19]. This increase in HVR seems to enhance exercise ventilation and improve SaO_2_ depending on the altitude where exercise is performed, since it was observed at 4500 m [14] but not at 2500 m or below [17], [20]. However, this difference could be due to the specific responses of the individuals of the different studies (i.e. low SaO_2_ values in [14]), and not necessarily to an effect of altitude per se. Additionally, IHE seems to affect metabolism during exercise [21] resulting in reduced LA during submaximal exercise and a rightward shift in the lactate-exercise curve [22]–[24]. Since lower LA is associated with a reduced ventilatory stimulus during exercise the IHE-induced metabolic adaptation may interact with the ventilatory adaptations at high altitude.

To our knowledge, no study focused on altitude-dependent effects of IHE on cardiorespiratory parameters during exercise and associated metabolic adaptations within the same subjects. Therefore, we determined the effects of IHE on cardiorespiratory and metabolic responses at different simulated altitudes in the same healthy subjects.

## Methods

### Ethics Statement

The study was carried out according to the Declaration of Helsinki and was approved by the Institutional Review Board of the Department of Sport Science (University Innsbruck). Written consent was obtained from all study participants after they were informed of the experimental procedures and possible risks involved with participation.

### Study protocol

**Figure 1:**
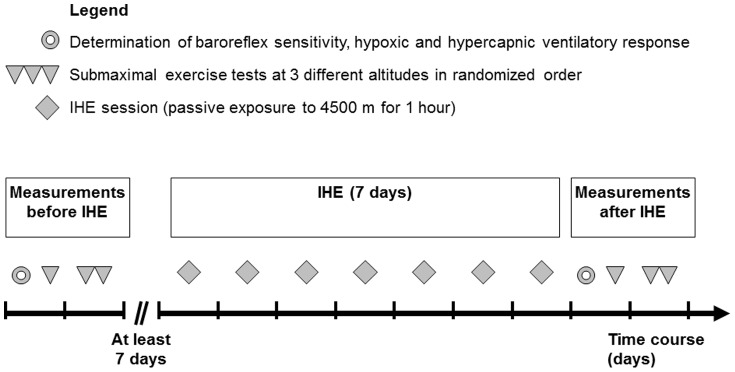
Time course of the measurements and the intermittent hypoxic exposure (IHE).

Eight healthy volunteers participated in the study (age 25±3 years, height 179±4 cm, body mass 74±5 kg). All participants were non smokers, had their residence below 800 m, and had no high-altitude exposures >2500 m during the previous 4 weeks. Each participant underwent a baseline examination. The study participants were instructed to perform no heavy exercise or high-intensity training sessions within the 2 days before the measurements. Measurements before IHE started with the determination of baroreflex sensitivity (BRS), HVR, and hypercapnic ventilatory response (HCVR). Subsequently, 4 submaximal exercise tests (in normoxia and at 3 different simulated altitudes) were conducted within 2 days. After a wash-out period of at least 7 days the IHE program started. The day after the last session of the IHE program the identical procedure started and measurements were completed at approximately the same time of day (±1 hour) as in the measurements before IHE. Thereby, the measurements after IHE took place at day 1 and 2 after finishing IHE (figure 1). The baseline examination, all measurements and the IHE program took place at the Department of Sport Science of the University Innsbruck (Austria) at an altitude of 600 m. After finishing all measurements and interventions of the study protocol the participants received a questionnaire to indicate the order of the simulated altitude, in order to assess the quality of the single-blinded study design.

### Baseline examination

Baseline examinations included a medical routine check, lung function testing and a maximal cycle ergometry. Lung function testing (Oxycon mobile, Jaeger, Germany) was performed in a sitting position on the cycle ergometer immediately before the ergometry to determine forced vital capacity (FVC) and forced expiratory volume in the first second (FEV_1_)). The better of 2 tests for FEV_1_ was taken for the evaluation. The cycle ergometry was performed on an electronically braked ergometer (Ergometrics 900, Ergoline, Germany) and started at a workload of 100 W for 5 minutes (warm up) with a following increase in workload of 25 W per minute until exhaustion. Heart rate and ventilatory parameters were monitored continuously (Oxycon mobile, Jaeger, Germany). Peak power output was defined as the last completed workload rate plus the fraction of time spent in the final uncompleted work rate multiplied by 25 W [20], [25]. Peak oxygen uptake (VO_2_peak) was defined as the highest 30-second average during the test. Results of the baseline examination are presented in table 1.

**Table 1:** Results of the baseline examination and calculated work loads for submaximal exercise testing.

Baseline examination	
Forced vital capacity (l)	6.1±0.6 (5.0–7.0)
Forced expiratory volume in the 1. second (l)	4.7±0.4 (4.2–5.4)
Peak power output (W)	337±33 (288–375)
Maximal heart rate (bpm)	193±6 (183–199)
Peak oxygen uptake (ml/min)	3805±462 (3211–4652)
Peak oxygen uptake (ml/min/kg)	51±7 (41–65)
Maximal ventilation (l/min)	163±24 (128–197)
Maximal respiratory exchange ratio	1.26±0.07 (1,15–1.38)

Calculated work loads for submaximal exercise testing	
40% of peak oxygen uptake (ml/min)	1527±170 (1284–1861)
Calculated work load (W)	99±13 (75–115)
60% of peak oxygen uptake (ml/min)	2290±255 (1927–2791)
Calculated work load (W)	176±19 (175–200)

Values are means ± SD (range).

### Measurements before and after intermittent hypoxic exposure

#### Baroreflex sensitivity and hypoxic and hypercapnic ventilatory response

All participants were tested in lying position at a comfortable temperature and humidity. Test equipment, procedure and data analysis were described and validated previously [26]–[28]. Briefly: The participants were connected to a rebreathing circuit through a mouthpiece. End-tidal carbon dioxide partial pressure (et-CO_2_; capnograph, COSMOplus, Novametrix, Wallingford, Conneticut, USA), oxygen saturation (pulse oxymeter, 3740 Ohmeda, Englewood, Colorado, USA) and air flow (Fleish pneumotachograph, Metabo, Epalinges, Switzerland) were measured continuously. Additionally electrocardiogram and non-invasive blood pressure were recorded continuously (Portapres, Finapres Medical Systems, Amsterdam, The Nederlands).

Arterial BRS was measured during 4 minute recordings on spontaneous breathing at each measurement session. From the original data the time series of RR interval (from each of 2 consecutive R waves of the electrocardiogram) and systolic blood pressure (SBP) were ob-tained. We used the average of a computed set of 7 different tests [29], as previous studies demonstrated poor correlation between different indices of BRS, and no method has shown clear superior performance over the other [30].

Subsequently, the participants were connected to the rebreathing circuit and the following rebreathing tests were performed: progressive normocapnic hypoxia (SaO_2_ from baseline to 80%, et-CO_2_ maintained at baseline level) and progressive hyperoxic hypercapnia (et-CO_2_ from baseline to +10–15 mmHg, SaO_2_ >98%). During the progressive isocapnic hypoxic test, the CO_2_ levels were kept at the desired level by passing a variable part of the expired air into a reservoir filled with soda lime, under continuous visual control of et-CO_2_. At the same time, by effect of rebreathing, the oxygen content of the rebreathing bag progressively decreased, hence inducing a reduction in SaO_2_. During the progressive hyperoxic hypercapnic test, oxygen was supplied to the rebreathing bag at very low flow, in order to maintain SaO_2_ above more than 98%, whereas the expired air was sent directly to the rebreathing bag inducing a progressive rise in et-CO_2_. The HVR and HCVR were obtained from the slopes of the linear regression of breath-by-breath minute ventilation vs. SaO_2_ or et-CO_2_, respectively.

#### Submaximal exercise tests

Submaximal exercise tests took place in a normobaric altitude chamber (Hypoxico Altitude Training Systems, Köln, Germany). The first test, which was performed under normoxic conditions, served for habituation to the test procedure and was not included into the analyses. The following 3 tests were conducted at 3 different simulated altitudes (altitude-1 to altitude-3). The order between the altitude-1 to altitude-3 was randomly balanced in a single-blind fashion to eliminate possible training and acclimatization effects by the testing procedure. Altitudes were simulated by reducing the inspired fraction of oxygen (F_I_O_2_) to the corresponding level at an altitude of 600 m (Innsbruck):
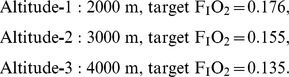



F_I_O_2_ and the inspired fraction of carbon dioxide (F_I_CO_2_) were controlled before, in the middle, and at the end of each test (Multiwarn II, Dräger, Lübeck, Germany). A carbon dioxide absorbing system in the altitude chamber lowered the increase in F_I_CO_2_ and thereby F_I_CO_2_ could be kept below 0.006. The 4 tests were completed within 2 days with 2 tests per day and a recovery period of at least 4 hours between tests.

After a 20-minute resting period in the chamber haematocrit and haemoglobin concentration were determined from capillary blood samples from the hyperaemized fingertip (Miniphotometer plus LP 20, Hach Lange, Düsseldorf, Germany). Subsequently, the submaximal exercise test was performed on an electronically braked cycle ergometer (Ergometrics 900, Ergoline, Bitz, Germany). The subjects cycled with a cadence of 70 rpm at 2 different work loads for 10 minutes each. The first work load (P_40_) corresponded to 40% and the second work load (P_60_) corresponded to 60% of VO_2_max determined during the baseline examination in normoxia (table 1). At P_40_ and P_60_, heart rate (Polar chest belt) and ventilatory parameters (Oxycon mobile, Viasys Healthcare, Höchberg, Germany) were monitored continuously. Steady state parameters of heart rate and ventilatory parameters were calculated as the means from minute 4:00 to 7:00 (3 minutes). During minute 7:00 to 8:00 capillary blood samples from the hyperaemized ear lobe were drawn to determine blood gases, pH (i-Stat Abbott, Birmingham, UK) and LA (Biosen C-line, EKF-diagnostic, Barleben, Germany). Bicarbonate concentration was calculated by the Henderson-Hasselbalch equation. SaO_2_ was measured by finger pulsoxymetry (Onyx 9550, Nonin, Plymouth, USA) during minute 8:00 to 9:00. Ambient temperature and humidity were controlled by a commercially available thermo-/hygrometer and kept between 21 and 25°C and 38 to 45%, respectively.

### Intermittent hypoxic exposure (IHE)

The IHE program comprised 7 resting sessions within 7 consecutive days (one session per day) each lasting 1 hour. During the sessions the participants were exposed to normobaric hypoxia (Hypoxico Altitude Training Systems, Köln, Germany) at a simulated altitude of 4500 m (F_I_O_2_ = 0.126). SaO_2_ (Onyx 9500, Nonin, USA) was measured after 30 minutes of each session. The subjects had no further altitude exposure >2500 m during the study period.

### Statistics

Statistical analyses were conducted by PASW Statistics 18 (IBM, Vienna, Austria). Normality in the distribution of data was tested by the Kolmogorov-Smirnov's test. Missing values for blood gases pH and bicarbonate concentration (n = 1–3) were replaced by group-average values at the specific altitude. Changes in BRS, HVR, and HCVR from before to after IHE were tested by paired students' t-tests. Differences in the measured variables among the experimental conditions were analyzed using two-way ANOVA with repeated measures (before – after IHE vs. 3 simulated altitudes). If overall significances for IHE or interactions were found, paired students' t-tests were carried out to find the source of difference. The relationships between variables were assessed by correlation analyses (Pearson or Spearman as adequate). P-values<0.05 (two-tailed) were considered to indicate statistical significance. Values are presented as means±SD.

## Results

All tests and the IHE program were well tolerated by the study participants without any side effects. Mean SaO_2_ during the IHE sessions varied from 81% during the 1^st^ session to 84% during the 7^th^ session but did not change significantly. The measured F_I_O_2_ values during the submaximal exercise tests before/after IHE at the 3 different simulated altitudes were 0.174±0.001/0.174±0.001 (2000 m), 0.154±0.000/0.154±0.001 (3000 m), and 0.135±0.001/0.135±0.001 (4000 m). The evaluation of the blinded study design revealed that no participant indicated the correct order of all simulated altitudes and only 38% of the indicated altitudes were correct.

**Figure 2:**
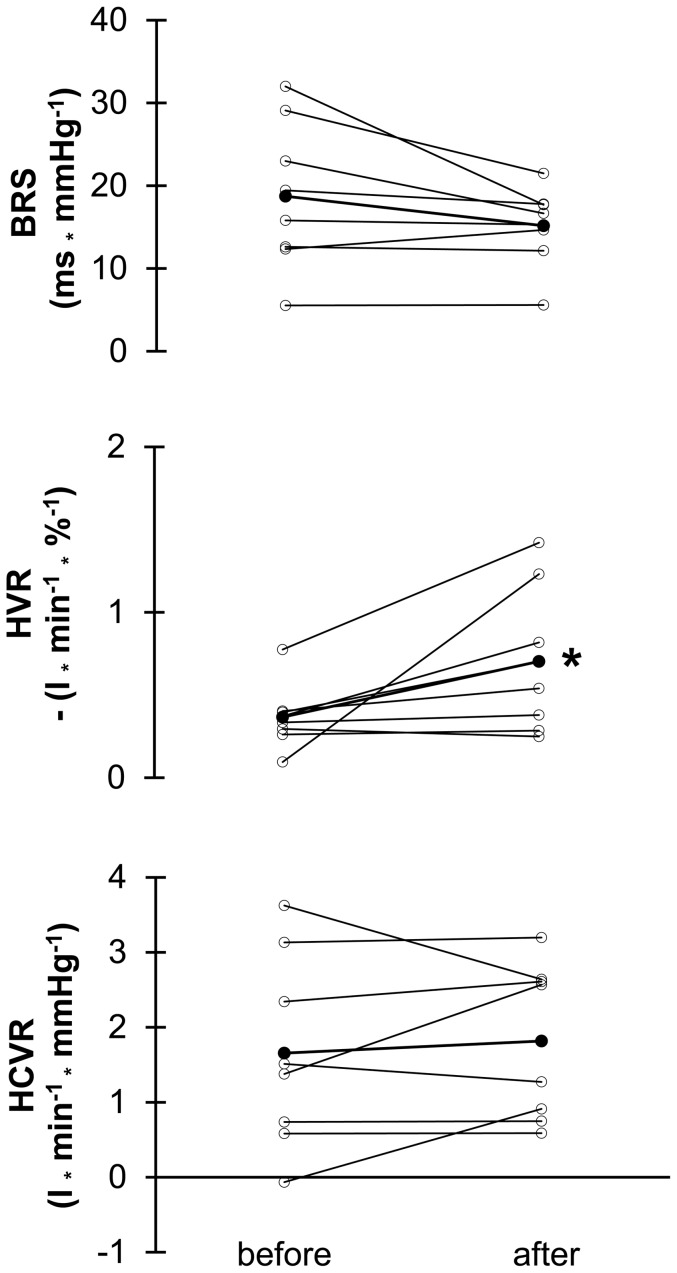
Baroreflex sensitivity, hypoxic and hypercapnic ventilatory response before and after intermittent hypoxic exposure. BRS = Baroreflex sensitivity, HVR = Hypoxic and ventilatory response, HCVR = Hypercapnic ventilatory response. Values are presented as individual values (thin lines) and means (thick line). * p<0.05 from before to after.

The applied IHE protocol resulted in a significant (p<0.05) increase in HVR whereas BRS (p = 0.11) and HCVR (p = 0.53) remained unchanged (figure 2). IHE had no effect on haematocrit (before vs. after: 45.9±2.1 vs. 44.9±1.9%, p = 0.08) and haemoglobin concentration (before vs. after: 15.5±0.9 vs. 15.4±0.5 mg*dl^−1^, p = 0.69).

**Figure 3:**
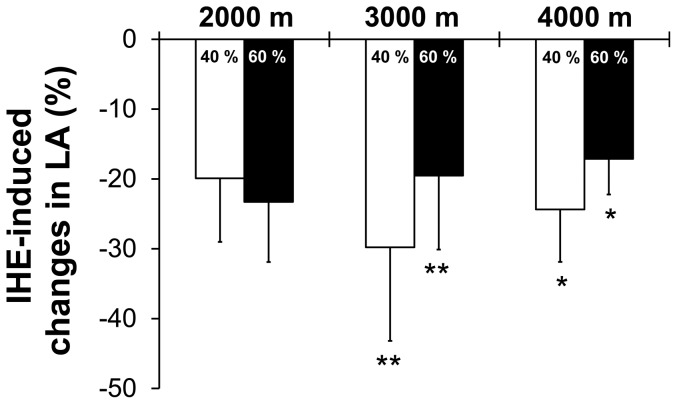
Changes in blood lactate concentration. Intermittent hypoxic exposure (IHE) induced changes (%) in blood lactate concentration (LA) at a workload corresponding to 40% and 60% of peak oxygen uptake in normoxia at different simulated altitudes (2000 m, 3000 m, and 4000 m). Values are means ± SEM. * p<0.05; ** p<0.01 (paired t-tests).

Results for the submaximal exercise tests at the 3 different simulated altitudes are shown in table 2 and table 3. Exposure to increasing altitudes induced the expected changes in the determined exercise responses at P_40_ and P_60_, such as significant increases in minute ventilation, breathing frequency, dead space to tidal volume ratio, alveolar ventilation, carbon dioxide output (VCO_2_), respiratory exchange ratio, ventilatory equivalents for oxygen and carbon dioxide, heart rate, LA, pH (only at P_40_) and significant reductions in SaO_2_, arterial partial pressures of oxygen and carbon dioxide and bicarbonate concentration. We found no significant interaction between the effects of altitude and those of IHE, indicating no altitude dependent effect of the applied IHE protocol. There were significant main effects of IHE on LA, VCO_2_, and partly on respiratory exchange ratio (p<0.05 at P_40_, p = 0.06 at P_60_). Post hoc analyses revealed significant reduction in LA at 3000 m and 4000 m (figure 3), significant reductions in VCO_2_ at 3000 m and for P_40_ at 4000 m. Fat oxidation at P_40_, calculated by the formula of Jeukendrup et al. [31], tended to be increased after IHE independent of altitude (p = 0.06). IHE did not influence minute ventilation, respiratory pattern (i.e. breathing frequency), ventilatory efficiency (i.e. dead space to tidal volume ratio), and alveolar ventilation but increased ventilatory equivalent for carbon dioxide. Post hoc analyses showed that this increase existed for P_40_ and P_60_ at 2000 m, 3000 m, and 4000 m. SaO_2_ improved slightly at P_60_ after IHE with no significant effects after post hoc testing. Blood gases (P_40_) and pH were generally affected by the IHE application. Post hoc tests revealed a significant increase in partial pressure for oxygen accompanied by a decrease in partial pressure for carbon dioxide at 3000 m and an increased pH at 2000 m (P_40_) and 4000 m (P_60_) after IHE.

**Table 2:** Cardiorespiratory parameters during submaximal exercise at different simulated altitudes before and after intermittent hypoxic exposure.

			2000 m	3000 m	4000 m	Altitude effect	IHE effect
**VE (l/min)**	P_40_	before	41±7	44±5	48±6	**	ns
		after	43±6	43±6	49±8		
	P_60_	before	72±12	77±12	87±11	**	ns
		after	74±12	77±13	91±15		
**BF (1/min)**	P_40_	before	20±7	20±7	21±7	**	ns
		after	21±8	21±7	23±8		
	P_60_	before	27±8	28±8	31±7	**	ns
		after	29±8	28±8	31±7		
**VD/VT (%)**	P_40_	before	18.0±2.1	20.1±2.3	21.0±3.3	*	ns
		after	18.4±1.7	19.9±2.4	21.0±2.0		
	P_60_	before	21.8±2.1	22.7±3.1	25.2±3.5	**	ns
		after	21.9±2.5	24.9±3.3	27.6±1.5		
**VA (l/min)**	P_40_	before	34±6	35±4	38±4	**	ns
		after	35±5	35±5	38±5		
	P_60_	before	57±8	59±8	65±8	**	ns
		after	58±10	58±9	66±10		
**VO_2_ (l/min)**	P_40_	before	1.58±0.22	1.58±0.19	1.61±0.19	ns	ns
		after	1.60±0.21	1.54±0.19	1.59±0.21		
	P_60_	before	2.53±0.33	2.56±0.30	2.47±0.19	ns	ns
		after	2.54±0.29	2.45±0.30	2.54±0.32		
**VCO_2_ (l/min)**	P_40_	before	1.42±0.20	1.44±0.17	1.51±0.17	*	**
		after	1.41±0.18	1.36±0.14§	1.44±0.17§		
	P_60_	before	2.38±0.24	2.43±0.25	2.48±0.28	*	*
		after	2.33±0.28	2.28±0.28§§	2.43±0.29		
**RER**	P_40_	before	0.90±0.03	0.91±0.03	0.94±0.04	*	*
		after	0.88±0.04	0.88±0.04	0.91±0.05		
	P_60_	before	0.94±0.04	0.95±0.04	1.00±0.05	**	ns
		after	0.92±0.05	0.93±0.05	0.96±0.06		
**VE/VO_2_**	P_40_	before	24.7±1.4	26.3±1.4	28.6±2.1	**	ns
		after	25.6±2.1	26.6±1.6	29.3±2.6		
	P_60_	before	27.5±1.8	28.8±2.0	34.1±2.5	**	ns
		after	28.1±3.2	30.4±2.9	34.7±4.4		
**VE/VCO_2_**	P_40_	before	27.5±1.8	28.9±1.5	30.5±2.3	**	**
		after	29.0±1.8§§	30.1±2.0§	32.2±2.0§§		
	P_60_	before	29.2±2.4	30.2±2.3	34.1±2.4	**	**
		after	30.7±2.3§	32.6±2.7§§	36.0±2.6§		
**HR (bpm)**	P_40_	before	115±7	118±8	124±9	*	ns
		after	114±10	116±9	119±9		
	P_60_	before	148±10	153±9	162±5	**	ns
		after	147±10	150±7	158±5		

IHE = intermittent hypoxic exposure,

P_40_/P_60_ = work load corresponding to 40/60% of peak oxygen uptake in normoxia, VE = minute ventilation, BF = breathing frequency, VD/VT = dead space to tidal volume ratio, VA = alveolar ventilation, VO_2_ = oxygen uptake, VCO_2_ = carbon dioxide output, RER = respiratory exchange ratio, VE/VO_2_ = ventilatory equivalent for oxygen, VE/VCO_2_ = ventilatory equivalent for carbon dioxide.

Values are means±SD.

Altitude effects = general effect (ANOVA) of the simulated altitude.

IHE effect = general effect (ANOVA) of IHE application (before vs. after) independent of simulated altitude.

There were no significant interactions (ANOVA) between simulated altitude and IHE.

ns p≥0.05,

* p<0.05,

** p<0.01.

§ p<0.05 and

§§ p<0.01 (post hoc) from before to after IHE at the specific altitude.

**Table 3:** Arterial oxygen saturation, blood lactate concentration, blood gases, and acid-base balance during submaximal at different simulated altitudes before and after intermittent hypoxic exposure.

			2000 m	3000 m	4000 m	Altitude effect	IHE effect
**SaO_2_ (%)**	P_40_	before	92.4±1.5	88.0±3.1	79.8±4.4	**	ns
		after	92.5±2.0	88.9±2.0	80.9±3.1		
	P_60_	before	89.8±2.1	83.3±2.9	77.3±3.8	**	*
		after	90.5±2.4	85.5±2.6	78.1±3.4		
**LA (mmol/l)**	P_40_	before	1.2±0.3	1.6±0.3	2.0±0.3	**	*
		after	0.9±0.3	1.0±0.3§§	1.5±0.5§		
	P_60_	before	3.5±1.1	4.1±1.4	6.3±1.3	**	*
		after	2.8±1.6	3.4±1.5§§	5.3±1.9§		
**PaO_2_ (mm Hg)**	P_40_	before	64.3±3.8	51.4±6.5	44.6±5.4	**	**
		after	64.1±4.3	55.6±3.2	45.9±3.8		
	P_60_	before	58.4±4.1	49.3±2.7	43.3±5.0	**	ns
		after	58.0±4.6	51.3±4.9	44.6±4.1		
**PaCO_2_ (mm Hg)**	P_40_	before	37.8±1.6	37.9±1.3	34.8±2.2	**	*
		after	37.2±2.2	36.2±1.5	34.2±1.6		
	P_60_	before	36.3±2.4	34.4±0.9	32.1±1.6	**	ns
		after	35.9±2.6	33.7±2.4	31.5±2.3		
**pH**	P_40_	before	7.44±0.02	7.44±0.01	7.47±0.03	**	*
		after	7.45±0.02§	7.45±0.02	7.46±0.02		
	P_60_	before	7.42±0.02	7.42±0.02	7.40±0.04	ns	**
		after	7.43±0.03	7.43±0.01	7.42±0.03§§		
**HCO_3_ (mmol/l)**	P_40_	before	24.49±1.32	24.80±0.79	24.40±1.67	*	ns
		after	25.34±2.27	24.63±1.65	23.78±1.55		
	P_60_	before	22.74±2.27	21.92±1.38	19.59±2.24	*	ns
		after	23.51±2.88	21.86±2.03	19.86±2.77		

IHE = intermittent hypoxic exposure,

P_40_/P_60_ = work load corresponding to 40/60% of peak oxygen uptake in normoxia,

SaO_2_ = arterial oxygen saturation, LA = blood lactate concentration (LA),

PaO_2_ = arterial pressure of oxygen, PaCO_2_ = arterial pressure of carbon dioxide, HCO_3_ = bicarbonate concentration.

Values are means±SD.

Altitude effects = general effect (ANOVA) of the simulated altitude.

IHE effect = general effect (ANOVA) of IHE application (before vs. after) independent of simulated altitude.

There were no significant interactions (ANOVA) between simulated altitude and IHE.

ns p≥0.05,

* p<0.05,

** p<0.01.

§ p<0.05 and

§§ p<0.01 (post hoc) from before to after IHE at the specific altitude.

**Figure 4:**
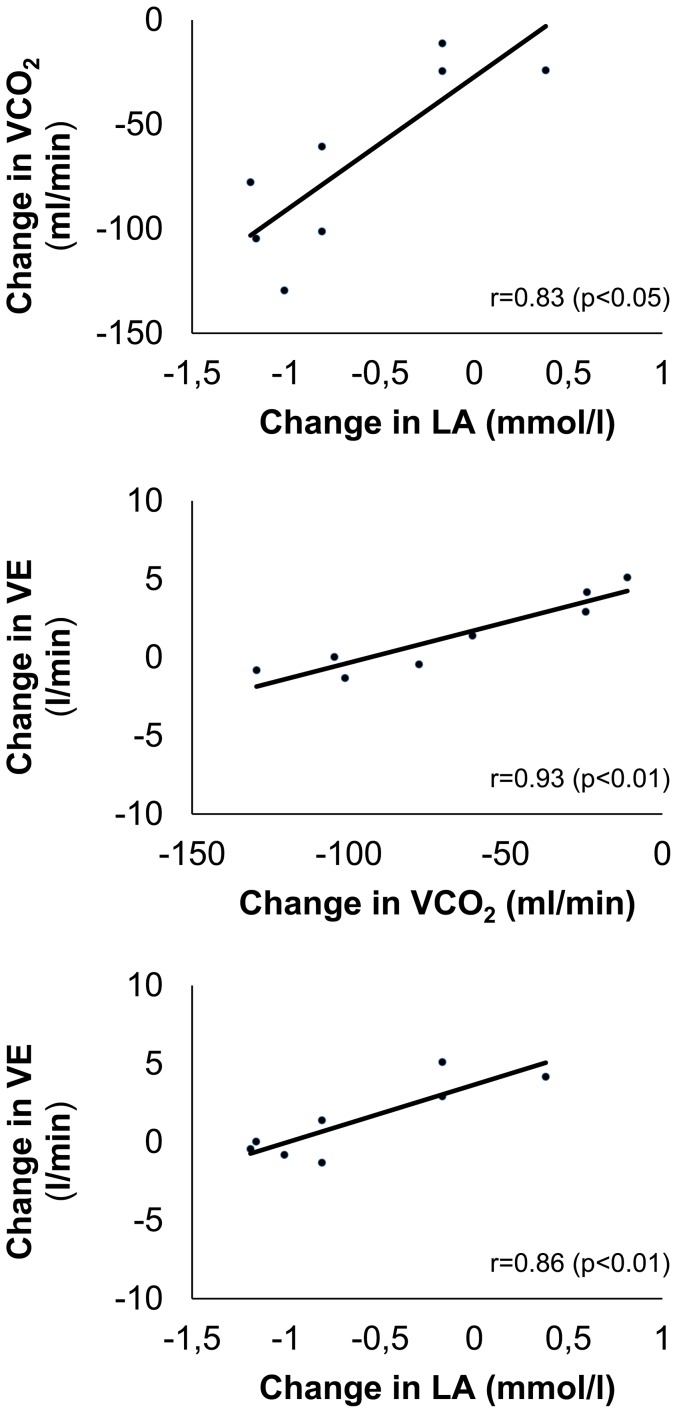
Relationship between blood lactate concentration, carbon dioxide output and minute ventilation. Relationship between the changes of blood lactate concentration (LA), carbon dioxide output (VCO_2_) and minute ventilation (VE) induced by intermittent hypoxic exposure. Dots represent individuals' mean changes at P_40_ and P_60_ at all 3 simulated altitudes were analysed.

Changes in HVR were associated with changes in LA (r = −0.72, p<0.05) and significant correlations for LA, VCO_2_, and minute ventilation occurred (figure 4) when individuals' means of P_40_ and P_60_ at all 3 simulated altitudes were analysed.

## Discussion

The main findings of our study were that the applied IHE protocol resulted in (1) reduced LA and VCO_2_ at 3000 m and 4000 m (2) no change in minute ventilation despite an enhanced HVR and (3) an increased ventilatory equivalent for carbon dioxide during exercise at all altitudes.

The observed changes in LA after IHE were general effects and irrespective of simulated altitude. The post hoc analyses revealed that the altitude-specific decreases of 17 to 30% were significant at 3000 m and 4000 m but did not reach significance at 2000 m (p = 0.11 and p = 0.09 for P_40_ and P_60_ respectively). Although the changes at 2000 m were in the same range as at 3000 and 4000 m, they were more heterogeneous. This was the reason for the non-significant results of the post hoc tests and may indicate that individual responses play a more important role at 2000 m compared to higher altitudes.

Reductions in submaximal LA after IHE have been repeatedly described under normoxic and hypoxic conditions and in subjects with various performance levels [20], [22]–[24]. For example, Burtscher et al. reported lower LA after IHE during submaximal exercise in normoxia in elderly subjects which were associated with an improved exercise tolerance [24]. In our study the IHE-induced changes in LA were associated with changes in VCO_2_ and minute ventilation. Since lactate acidosis, caused by H^+^-ion production equimolar with lactate production, and VCO_2_ are strong stimuli of exercise ventilation [32], the contribution of LA and VCO_2_ to the ventilatory drive was lower after IHE.

The reasons for the observed reduction in submaximal LA remain speculative. They might have been caused by an improved maximal aerobic power resulting in lower relative exercise intensity after IHE. However, based on our data it is not likely that aerobic power was improved because neither haematological parameters (i.e. haemoglobin concentration) nor heart rate as an indicator of relative exercise intensity had changed after the IHE application. However we cannot entirely exclude changes in aerobic power because we did not determine VO_2_peak after the IHE intervention.

Alternatively, the reduction in LA could be a result of an increased fat utilisation after IHE. This assumption is supported by the lower respiratory exchange ratio and the calculated fat oxidation rates at P_40_ which were increased by about 30% (average of all 3 simulated test altitudes) after IHE. Since mitochondrial function seems to be largely unaffected by high-altitude acclimatization [33] this effect might be caused by an improved muscular oxygen availability and a lesser beta-adrenergic stimulation of glycolysis after IHE [24], [34]–[36]. However, the unaltered BRS after IHE, indicating no changes in sympathetic activity, are unexpected and in contrast to results in COPD patients [28]. However, COPD patients presented a depressed BRS pre-intervention whereas our subjects had normal BRS values without further improvement. Additionally, BRS measurements we performed under normoxic conditions and cannot be generalized to hypoxic conditions were exercise was performed.

The significant negative correlation between changes in HVR and in LA and the slightly improved SaO_2_ suggest the following mechanism. The increased HVR resulted in an improved arterial oxygenation (SaO_2_) which is tightly coupled to an improved muscular oxygen availability resulting in an increased fat oxidation rate and lower blood lactate levels. However, since we did not provide data of muscular blood flow before and after IHE, the link between arterial and muscular oxygenation could not be proven by our data.

An increased fat utilization should result in a higher oxygen uptake at the same workload which was not the case in our study. Although there is strong evidence that acclimatization to moderate to high altitude does not alter exercise economy [37], repeated short-term (≤3 hours) exposures to hypoxia have been shown to improve exercise economy [38], [39]. This effect may have masked the increase in oxygen consumption caused by the higher fat utilization.

The increased HVR and the unaffected HCVR are in agreement with other studies using short-term IHE [15]–[19] and indicate a more pronounced chemosensitivity to hypoxia accompanied by an unaltered chemosensitivity to hypercapnia. However, this increased hypoxic chemosensitivity did not result in increased minute ventilation, breathing frequency, alveolar ventilation and SaO_2_ during exercise at 2000 to 4000 m simulated altitude. Although our observations are partly supported by studies reporting no IHE-induced changes in exercise ventilation and SaO_2_ at altitudes up to 2500 m [17], [20] we expected significant effects above 2500 m [14] or at least an altitude-dependent effect indicated by a significant interaction between the IHE and simulated altitude.

Although the reasons for this discrepancy cannot be explained by our data several factors can be discussed. Mean SaO_2_ values of our subjects at a simulated altitude of 4000 m were 80% at P_40_ and 77% at P_60_ whereas SaO_2_ values in the study of Katayama et al. were below 65% at 40% and below 60% at 70% of VO_2_peak at a simulated altitude of 4500 m [14]. As a consequence the stimulus to the peripheral oxygen sensing chemoreceptors was markedly lower in our subjects and could have contributed only slightly to the regulation of exercise ventilation compared to other stimuli (e.g. LA). Therefore it might be possible that the changes in chemosensitivity to hypoxia, i.e. the increase in HVR, were not sufficient to modify exercise ventilation in our subjects.

Furthermore, the observed reductions in LA and VCO_2_ may have counteracted the increased HVR resulting in no significant net effect on minute ventilation. This assumption is supported by (1) the statistical association between changes in LA, VCO_2_ and minute ventilation and (2) the observed increase in the ventilatory equivalent for carbon dioxide, which is considered as an index of global chemosensitivity [40]. The increase of the global chemosensitivity, which resulted in a relative (in relation to carbon dioxide output) hyperventilation, must be caused by the isolated HVR increase because HCVR did not change. When combined with the reduced carbon dioxide output it is explainable that exercise ventilation was not influenced by the applied IHE protocol despite an increase in HVR.

From a practical point of view it would be interesting to see whether the observed metabolic and cardiovascular adaptations would result in improved endurance performance. Due to the chosen study design it was not possible to evaluate endurance performance at the different altitudes in this study and therefore the question remains open if the detected reductions in LA would result in an improved endurance performance. On the one hand IHE seems not to improve 30-minute time-trial performance at 1970 m in a double-blind placebo controlled study design despite reduced blood lactate concentrations [20]. On the other hand IHE (7×3 hours at 4300 m simulated altitude) had positive effects on endurance performance only when tested the day after the last IHE session [12], [41]. However, up to now no study tested IHE protocols using passive exposures ≤1 hour with respect to endurance performance at altitudes >2000 m.

At least 2 limitations of the presented study results have to be addressed. First, the relative small sample size resulted in a low statistical power for the non-significant changes. Therefore it might be possible that existing changes where not detected in our study. Second, we tested the pre-acclimatization effects of repeated exposures to only 1 altitude, i.e. 4500 m. Therefore, the results cannot be generalized to other IHE protocols with lower or higher hypoxic stimuli.

In conclusion, the applied IHE protocol provoked metabolic changes (i.e. reduced LA), which seem to interact with IHE-induced respiratory adaptations (i.e. increased HVR) during submaximal exercise at simulated altitudes between 2000 m and 4000 m. This effect occurred independent of simulated altitude. Future studies should investigate if IHE-induced changes improve endurance performance and should address the comparison of different IHE protocols.
